# Non-syndromic bile duct paucity and non-IgE cow’s milk allergy: a case report of challenging nutritional management and maltodextrin intolerance

**DOI:** 10.1186/s13052-022-01358-8

**Published:** 2022-09-15

**Authors:** Irene Degrassi, Martina Chiara Pascuzzi, Enza D’Auria, Laura Fiori, Dario Dilillo, Gianluca Lista, Francesca Maria Castoldi, Francesco Cavigioli, Alessandra Bosetti, Alessandro Pellegrinelli, Gian Vincenzo Zuccotti, Elvira Verduci

**Affiliations:** 1 Department of Pediatrics, “Vittore Buzzi” Children’s Hospital, Milan, 20154 Italy; 2 Department of Pediatrics, Neonatal Intensive Care Unit, “Vittore Buzzi” Children’s Hospital, Milan, 20154 Italy; 3 Department of Pathology, “L. Sacco” Hospital, Milan, 20157 Italy; 4 Department of Biomedical and Clinical Science, University of Milan, Milan, 20157 Italy; 5 Department of Health Sciences, University of Milan, Milan, 20142 Italy

**Keywords:** Cholestasis, Premature infants, Bile duct paucity, Maltodextrin, Hypoglycaemia

## Abstract

**Background:**

Cholestasis in extremely premature infants (EPI) constitutes a nutritional challenge and maltodextrins have been reported as a possible strategy for hypoglycaemia. We aim to describe the nutritional management of an EPI with non-syndromic bile duct paucity (NSBDP) and feeding intolerance.

**Case presentation:**

A patient, born at 27 weeks of gestational age, presented cholestatic jaundice at 20 days of life with a clinical picture of NSBDP. Patient’s growth was insufficient with formula rich in medium-chain triglyceride (MCT) and branched-chain amino acids (BCAA). Due to frequent fasting hypoglicemic episodes, maltodextrins supplements were provided. He subsequently presented severe abdominal distension and painful crises, which required hospital admission and withdrawal of maltodextrins. Hypercaloric extensively hydrolysed formula provided weight gain, glycemic control, and parallel improvement in cholestasis**.**

**Conclusions:**

Our case suggests caution with the use of maltodextrins in infants, especially if premature. Commercial preparations for hepatopatic patients contain higher concentrations of MCTs and BCAAs, but personalized strategies must be tailored to each patient.

## Background

Cholestasis in preterm infants has multifactorial etiology. Several factors have been associated with this condition in Neonatal Intensive Care Unit (NICU) such as sepsis, gastrointestinal surgery, drug toxicity, delay in starting of enteral feeding and prolonged parenteral nutrition. Extremely low birth weight infants has been reported at risk of more severe forms of cholestasis with consequent malnutrition, poor brain development and neurocognitive outcomes [1].

Nutritional management is therefore essential to improve the outcome of these patients, but can be challenging due to high energy requirement and malabsorption of lipids and fat-soluble vitamins.

Several strategies to maintain glucose and reach calories targets have been proposed including the use of maltodextrins, but safety among infants and premature newborns are still unclear [2].

Non-syndromic bile duct paucity (NSBDP) represent a rare cause of cholestasis, diagnosis is based on histology associated with clinical features, it can occur up to 20% in preterm infants and its phenotypic spectrum has not yet been well described [3].

We aim to describe the challenging nutritional management and maltodextrin intolerance in a rare case of an extremely premature infant with non syndromic bile duct paucity.

## Case presentation

A twenty-five weeks old infant (12 weeks of correct age) was admitted to our Paediatric Emergency Department (PED) for inconsolable crying, abdominal distension, diarrhoea, feeding and growth failure.

He had been delivered in our NICU by urgent caesarean section, from non-consanguineous healthy parents, at 27 + 5 weeks’ gestational age from a spontaneously occurring monochorionic triamniotic trigemellar pregnancy complicated by intrauterine growth retardation (IUGR) of two twins. His family history was known for gallstones (twin brother, maternal grandmother and paternal grandfather) and Hashimoto's thyroiditis (mother). The Apgar score at birth was 6/9. His birth weight was 690 g (9th percentile), length 32 cm (6th percentile) and cranial circumference 23.8 cm (15th percentile). Minimal enteral feeding was provided concurrently with parenteral nutrition from birth. Parenteral nutrition was progressively tapered off until full enteral feeding was achieved at 26 days of age. From 20 days of life cholestatic jaundice (total serum bilirubin 15.31 mg/dl, direct serum bilirubin 4.21 mg/dl), with normal gamma-glutamyl transferase (GGT) and hypocholic stools was observed.

**Fig. 1:**
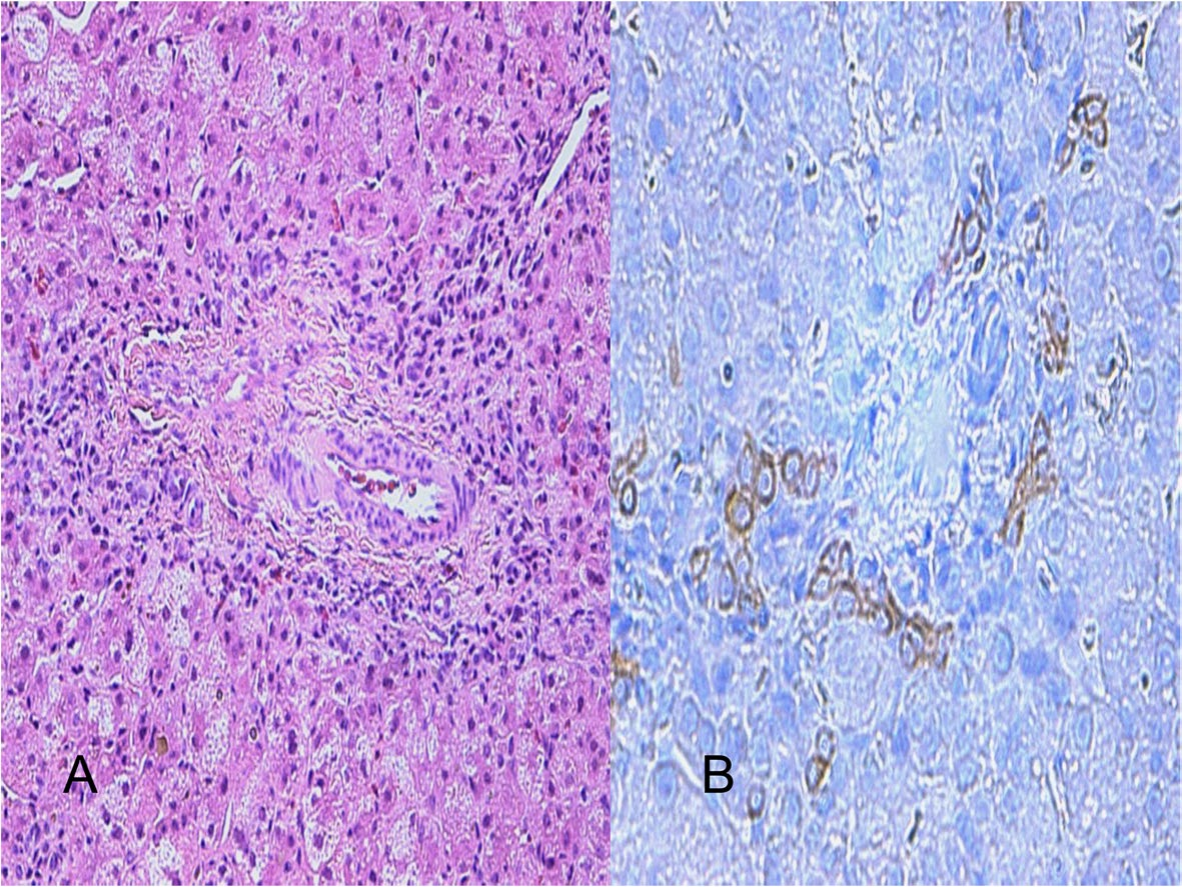
**A** Portal tract without bile duct, balloning changes of hepatocytes and canalicular cholestasis( EE, 20x). **B** Weak ductular reaction ( cytocheratin 7 immunohistochemestry, 40 x)

Diagnosis of NSBDP was based on exclusion of anatomical, genetic, endocrine, infectious and metabolic causes of cholestasis. These included serologic tests for intrauterine infections, alfa-1-antitrypsin levels, serum and urine amino acids, urine organic acids, levels of very long chain fatty acids, qualitative urine bile acids analysis, isoforms of transferrin, screening test for cystic fibrosis and lysosomopathies. Liver biopsy showed intracanalicular cholestasis with absence of bile ducts in portal tracts within twelve portal spaces (Fig. 1A), modest ductular reaction (Fig. 1B). No evident inflammatory or necrotic component, and absence of fibrosis. Deposits of PAS-d positive material were not noticed in hepatocyte cytoplasm. Whole exome sequencing was negative and intraoperative cholangiography were performed showing regular opacification of the bile ducts. Vertebral, ocular and cardiac anomalies were excluded. The clinical course was also complicated by severe bronchopulmonary dysplasia (sBDP) and grade I intraventricular haemorrhage (IVH). Patient growth was poor with whole protein formula (86,4 kcal/100 mL) recommended for cholestasis rich in medium-chain triglyceride (MCT) (up to 50%) and branched-chain amino acids (BCAA) (up to 30%), started at 13 weeks of post-natal age, which provided approximately 130% of the caloric requirements for age. Due to frequent fasting hypoglycemic episodes, nocturnal enteral feeding with nasogastric tube was introduced. At 23 weeks of life (9 weeks of corrected age) oral maltodextrin supplements (Fantomalt), a product protein, lactose and fiber free, were provided. The infant had been discharged from our NICU, in a satisfactory condition, on the 25st weeks of life (12 weeks of correct age). After two weeks since the introduction of maltodextrins he presented abdominal distension and crying crises so he was admitted to paediatric ward. At admission, physical examination was unremarkable, except for cutaneous-scleral jaundice and painful, distended and meteoric abdomen. Hypocolic stools were also observed. Routine blood tests confirmed cholestatic jaundice with increased alanine transaminase (ALT) and normal GGT values (total serum bilirubin 12.5 mg/dl, direct serum bilirubin 9.0 mg/dl, ALT 534 U/L). Abdominal ultrasound scan (US) revealed a modestly enlarged liver with an inhomogeneous ecostructure, a normal intrahepatic and extrahepatic biliary tree and ileal loops distended by gaseous material. Abdominal X-ray ruled out free air in the abdomen and surgical urgencies. Diarrhoeic discharges after meals and poor fasting tolerance were observed during hospitalisation, so gastrointestinal transit assessment showed normal gastric emptying time and regular disposition of bowel loops. Oral maltodextrins supplementation has been interrupted and, due to family history (twin brother) of non-IgE mediated cow's milk allergy, an allergological evaluation was perfomed and search for sIgE to cow’s milk proteins, which resulted negative. A hypoallergenic casein based extensively hydrolysed formula, rich in MCT (55% of total lipids) (0,66 kcal/ml) was started with prompt remission of symptoms. Due to low energy intake, poor growth and episodes of hypoglycaemia, reintroduction of 2 gr after every meal (10 gr per day) of maltodextrins for 24 h was attempted. Crying crises and diarrhoea immediately reappeared, so maltodextrins administration was definitively interrupted and only hypercaloric seroproteins extensively hydrolysed formula (1 kcal/ml) enriched with high percentage of MCT (48%) was introduced. Dietary tolerance was then observed, so the energy intake of enteral nutrition with extensively hydrolysed formula was increased to meet total daily energy requirements. Recovery of weight gain and improvement in glycaemic control were observed, with parallel improvement in cholestasis. Ursodeoxycholic acid therapy and fat soluble vitamin (ADEK) was administered throughout the hospitalization.

At discharge (7 months of age, 5 months of corrected age) total serum bilirubin was 2.3 mg/dl, with direct serum bilirubin 1.6 mg/dl. No abdominal pain crises have been reported since the discontinuation of maltodextrins.

## Discussion and conclusions

Cholestasis is defined as a reduced flow of bile leading to a decreased concentration of bile acids in the intestinal lumen and accumulation of bile substances in the blood. It affects about 1 of 2500 newborns and is most commonly reported in preterm infants [4]. Tufano et al. [5] showed 27 cases of cholestasis with 92.5% premature infants in a cohort of 1289 infants admitted to NICU, 80% of whom had a birth weight < 1000 g. Our case shows that, despite being a frequent findings in NICU, the diagnostic course can be prolonged and can require several invasive exams. Furthermore, frequent complications are malnutrition and macro-micronutrient deficiency due to increased energy expenditure, maldigestion of lipids and fat-soluble vitamins, diarrhoea, peripheral lipolysis and suboptimal protein synthesis in the liver and muscle [6]. The presence of cholestasis can lead to worsening of the prognosis and outcome in such complex patients. Niccum et al. [1] demonstrated a higher risk of cholestasis in preterm infants having lower Apgar scores and lower birth weight, in correlation with decreased weight percentile at discharge with subsequent impact on neurological outcome. Although cholestasis is typically multifactorial and transient, prognosis can be variable and often poor. Our patient's monochorionic twin brother presented higher birth weight and milder and transient cholestasis, he had hospitalization shorter than five months, better weight catch-up and neurological outcome.

The diagnosis of intrahepatic bile duct paucity (PIBD) is mainly histological: defined as loss of at least 50% of bile ducts in ten portal spaces, with correlation to clinical and biochemical findings [3]. Classically there are two forms: syndromic (associated with Alagille syndrome and arthrogriposis renal dysfunction and cholestasis—ARC syndrome) and non-syndromic secondary to chromosomal, infectious, inborn error of metabolism, endocrinological, myeloproliferative or idiopathic causes [3]. Studies have shown that the bile ducts/portal tracts (BD/PT) ratio progressively increases in the postnatal period during the first weekes of life, making difficult to diagnose PIBD before 38 weeks of postconceptional age [7]. The clinical presentation of our patient was similar to that of the series reported in the literature (Table 1) with jaundice, acholic stools and growth retardation, he showed resolution of cholestasis at 8 months of age.

**Table 1:** Summary of reported cases series of NSPBD

**Publications**		**N**	**Prematurity**	**Age, mo**	**Sex**	**Jaundice**	**Pale stool**	**Hepatomegaly**	**Splenomegaly**	**Growth failure**	**Poor outcome**
Bruguera et al., 1992 [8]		4	0/4	5—144	F > M	2/4	not reported	4/4	2/4	not reported	1 D 1 C
Chiu et al., 1996 [9]		4	not reported	0—132	M > F	4/4	not reported	4/4	2/4	4/4	3 D
Koçak et al., 1997 [10]		10	1/10	0.5 – 36	M > F	10/10	7/10	8/10	7/10	5/10	3 D
Yehezkely-Schildkraut et al., 2003 [11]		10	2/8	0 – 6	M > F	10/10	4/10	5/10	not reported	not reported	2 LF 4 UC
De Tommaso et al., 2004 [12]		11	2/11	1 – 40	M > F	11/11	8/11	6/11	4/11	1/11	1/11 C + LT
Meena et al., 2018 [3]	Non-destructive PILBD	20	7/20	4 – 12	M > F	18/20	10/20	20/20	19/20	11/20	3 LT
	Destructive PILBD	50	8/50	4 – 97	M > F	48/50	23/50	49/50	47/50	22/50	26 LT
Mohanty et al., 2019 [13]		18	1/18	1 – 11	M > F	3/18	12/18	2/18	7/18	50–90%	1 D

*N* Number of patiens, *Mo* Months, *M* Male, *F* Female, *D* Death, *LF* Liver failure, *R* Renal disease, *U* Unchanged cholestasis, *C* Cirrhosis, *LT* Liver transplantation

A second liver biopsy was not done in our patient, but the clinical trend suggests that growth led to an improvement in hepatic and biliary damage. Meena et al. [3] reported that some factors such as intrauterine failure to thrive or sepsis may lead to PIBD in premature infants due to interference with the developing ductal system. It could be that prematurity constitutes a spectrum of this pathology with its characteristics and prognosis, future studies will be needed to analyze this particular subpopulation of patients in more detail. Besides, the prognosis and progression of NSPBD have been widely debated. Past studies reported the non-syndromic forms of NSPBD as the type with the worst prognosis [3]. Chiu et al. [9] reported that one patient of 4 non-syndromic patients survived, during follow-up he presented delayed growth and became jaundice-free after the age of 5 years. In the cohorts analysed (Table 1), only 8 cases reported fatal outcome, but frequent and severe complications such as liver and kidney failure, chronic liver disease and cirrhosis were described and can define the poor prognosis of this condition. Indeed, the belief that the non-syndromic variety progresses more towards cirrhosis than the syndromic variety [14], has been recently contested [13]. Associated familial/metabolic conditions may influence morbidity, mortality and prognosis [11]. Diagnosis of non-syndromic form should not be considered as unfavorable prognosis, as the evolvement should be variable and related to the etiology in this form of presentation [8, 10, 12].

Despite different nutritional strategies and complete coverage of energy requirements, our case, born with severe intrauterine and postnatal growth retardation, continue to present poor weight gain and abdominal symptoms. Strategies adopted included increasing meal frequency and feeding volume, supplementing with medium-chain triglycerides, using specific formulas based on whole milk proteins with BCAAs, intended for hepatopathic patients and supplementing with fat-soluble vitamins. Struggling with hypoglycaemia and poor growth, short-chain polymer (maltodextrin) supplementation has been proposed at 23 weeks of postnatal age (9 weeks of corrected age). Unfortunately, after few weeks our patient presented severe pain crises which required hospitalization, so that different dietary modifications were performed to achieve food tolerance. Maltodextrins were initially discontinued, but abdominal symptoms persisted until the formula was changed with casein based extensively hydrolysed formula, with high percentage of MCT (55% of total lipids). Afterwards maltodextrins were reintroduced, while maintaining a diet free of milk proteins, diarrhoea, bloating and abdominal pain promptly reappeared.

Carbohydrate supplementation such as maltodextrins and starch have been proposed as possible alternatives in patients with cholestasis at high risk of hypoglycaemia, also taking into account that the increase in lipid supplementation is limited due to poor absorption [15]. However, studies in preterm animals have shown that supplementation with maltodextrins leads to alterations in the bacterial flora with production of gas and metabolites such as short-chain acids resulting in an increased risk of necrotizing enterocolitis (NEC), currently, no studies have investigated efficacy and safety of maltodextrins in infants and older children [16] and an adverse reaction had never been described in this population. It should be considered that severe abdominal bloating and diarrhea can occur, because of maldigestion due to the enzymatic immaturity of amylases at an early age [2].

In our patient persistence of adbominal symptoms after maltodextrins discontinuation and symptoms relief with the introduction of extensively hydrolyzed based formula are suggestive of a non-IgE mediated cow milk allergy (CMA), more than maldigestion. However, our case has some limitations: the main one is that an oral food test was not performed to confirm the diagnosis of non-IgE CMA, as the parents refused to do it [17]. The second limitation was that intake of different sources of maltodextrin have not been studied.

Our description reports a rare case of non-syndromic bile duct paucity. To our knowledge, this is the first case reported of maltodextrins intolerance in a preterm infant, complicated by a non-IgE mediated cow-milk allergy, which made the nutritional management even more difficult to deal with and interpret. Our case suggests the importance of a multidisciplinary approach and possible switch to an hypoallergenic formula, based on extensively hydrolyzed proteins, in case of symptoms persistence with the use of whole protein based formula intended for cholestasis. Moreover, we suggest caution in supplementing with carbohydrates infants and careful monitoring adverse reaction during the dose increase for possible complication. There are several gaps and lack of studies on the efficacy of different strategies in paediatric patients with cholestasis, also regarding maltodextrin supplementation. Commercial preparations for hepatopatic patients contain higher concentrations of MCTs (30–50%) and BCAAs, but personalized strategies must be tailored to each patient based on comorbidities, clinical response, and specificrequirements.

## Data Availability

The datasets used and/or analysed during the current study are available from the corresponding author on reasonable request.

## Abbreviations

ALT: Alanine transaminase
ARC: Arthrogriposis renal dysfunction and cholestasis
BCAA: Branched-chain amino acids
BD/PT: Bile ducts/portal tracts
CMA: Cow-milk allergy
EPI: Extremely premature infants
GGT: Gamma-glutamyl transferase
IUGR: Intrauterine growth retardation
IVH: Intraventricular haemorrhage
MCT: Medium-chain triglyceride
NEC: Necrotizing enterocolitis
NICU: Neonatal intensive care unit
NSBDP: Non-syndromic bile duct paucity
PED: Paediatric Emergency Department
PIBD: Intrahepatic bile duct paucity
sBDP: Severe bronchopulmonary dysplasia
US: Ultrasound scan
